# Expression of E-cadherin in Hyperplastic Endometrium: A Retrospective Analysis

**DOI:** 10.7759/cureus.65225

**Published:** 2024-07-23

**Authors:** Monisha Rita Jayaraman, Niveditha EN, Volga Harikrishnan

**Affiliations:** 1 Pathology, Saveetha Medical College, Saveetha Institute of Medical and Technical Sciences, Saveetha University, Chennai, IND

**Keywords:** premalignant conditions, hyperplastic endometrium, cell adhesion molecule, atypical endometrial hyperplasia, endometrial hyperplasia, e-cadherin

## Abstract

Aim

Epithelial cadherin or E-cadherin is a cell adhesion molecule that is present in all cells to promote integrity and survival of the cells. The aim of this study was to assess the immunohistochemical staining pattern of E-cadherin in hyperplastic endometrium.

Methods

A total of 25 blocks of formalin-fixed paraffin-embedded tissues of endometrial biopsies, from September 2020 to May 2023, were obtained from the Department of Pathology, Saveetha Medical College. Out of these 25 histologically proven cases of endometrial hyperplasia (EH), 17 cases were of EH without atypia and 8 cases were of endometrial hyperplasia with atypia (AH, or atypical hyperplasia).

Results

The immunohistochemical examination revealed that E-cadherin expression was downregulated in both EH without atypia and AH. But the downregulation was more pronounced in cases of AH than in EH without atypia. This was confirmed by the comparison of E-cadherin expression between EH with and without atypia by a chi-square test, which showed a p-value of 0.05 and was proven significant.

Conclusion

The heterogeneous expression of E-cadherin can be attributed to the impairment of cadherin-catenin complex. This impairment is seen in AH as well as EH without atypia. This shows this impairment occurs very early in the transformation process of the endometrium from hyperplastic to neoplastic.

## Introduction

Endometrial hyperplasia (EH) is a precursor lesion of endometrial carcinoma. EH is the proliferation of endometrial glands due to the unopposed action of estrogen [1]. The effects of estrogen are normally counterbalanced by the effects of progesterone, and when not present results in unchecked proliferation of the endometrium. EH is characterized by the increase in the gland-to-stromal ratio of the endometrium, which is normally 1:1 [1,2]. It is classified based on the presence of atypia as EH without atypia and atypical hyperplasia (AH) [1,2]. There are many overlapping features between AH and endometrial carcinoma (EC) both clinically and histopathologically [2]. In this study, we use E-cadherin, a cell adhesion molecule, and assess its staining pattern in EH with the help of immunohistochemistry (IHC). E-cadherin promotes intercellular adhesion and integrity with interactions with actin cytoskeleton [3]. It mediates interactions with some cytoplasmic domains and forms the cadherin-catenin complex. Any alteration in E-cadherin expression impairs the cadherin-catenin complex [3]. The significance of E-cadherin expression in hyperplasia is discussed further in this article.

## Materials and methods

This study was conducted at Saveetha Medical College, Chennai. Relevant clinical details such as patient age, clinical presentation, parity, and endometrial thickness for the biopsy specimens were obtained from the requisition forms sent by the clinician. For total abdominal hysterectomy specimens, gross measurements of endometrial thickness were collected. Paraffin blocks of tissues fixed in 10% neutral buffered formalin were obtained and trimmed. An approval was obtained from the Institutional Ethics Committee (no. 223/06/2024/PG/SRB/SMCH).

Three- to four-micron sections were taken from each block using a Leica 2125RTS manual rotary microtome (Leica Biosystems, Deer Park, IL). The sections were mounted on egg albumin-coated, labeled glass slides and air-dried. The slides were kept in a staining rack and stained with Harris hematoxylin and eosin (H&E) using a Leica Auto Stainer XL (ST5010; Leica Biosystems). H&E-stained sections were examined to check the adequacy of the tumor components. Blocks with adequate material were selected for further study. Three- to four-micron sections were taken from each block using a Leica 2125RTS microtome. The sections were mounted on H&E-labeled slides and stained by Harris H&E. Once the slides were dry, they were stored in a tray according to study numbers. The cases were evaluated by the principal investigator, and the information was recorded on the datasheet. For immunohistochemistry, two- to four-micron sections were cut from each study block and mounted on two poly-L-lysine treated slides labeled with lab number and type of test. When dried, they were stored in a tray and processed in different batches. One positive and one negative control were included in each batch and subjected to the same test conditions as the study cases. Manual immunostaining was performed after mounting the sections on poly-L-lysine-treated slides. E-cadherin rabbit monoclonal antibody clone EP6 was used as the primary antibody for immunohistochemical staining. The secondary antibody used was the PolyExcel HRP/DAB detection system (PathnSitu Biotechnologies, Hyderabad, India), a two-step universal kit for mouse and rabbit primary antibodies.

The interpretation of immunohistochemistry followed a previous study [4]. For each case, at least 10 low-power fields (10x) were examined. The stained IHC slides were analyzed independently by two pathologists to avoid inter-observer variations. The presence of reaction (indicated by brownish-colored products), cellular localization (nuclear/membrane/cytoplasm), and percentage of cells stained were evaluated. Strong intermembranous staining of E-cadherin with absent cytoplasmic positivity was considered positive. Aberrant staining of E-cadherin, such as weak discontinuous membranous staining or membrano-cytoplasmic staining or perinuclear dot-like staining, was considered negative. Scores of 0, 1, 2, and 3 were given, as shown in Table 1. If more than 75% of the cells stained positive, a score of 3 was given. If 51%-75% of the cells stained positive, a score of 2 was given; if 6%-50% stained positive, a score of 1 was given; and if less than 5% of the cells stained positive, a score of 0 was given. A score of 3 indicated normal E-cadherin expression. Scores of 1 and 2 indicated heterogeneous expression due to downregulation, and a score of 0 indicated no expression of E-cadherin.

**Table 1:** Scoring system used to interpret of E-cadherin expression with immunohistochemistry

Percentage of cells staining positive	Score	Staining pattern	E-cadherin expression
>75	3	Homogeneous	Normally expressed
51-75	2	Heterogeneous	Downregulated
6-50	1	Heterogeneous	Downregulated
<5	0	Negative	Absent

## Results

**Figure 1:**
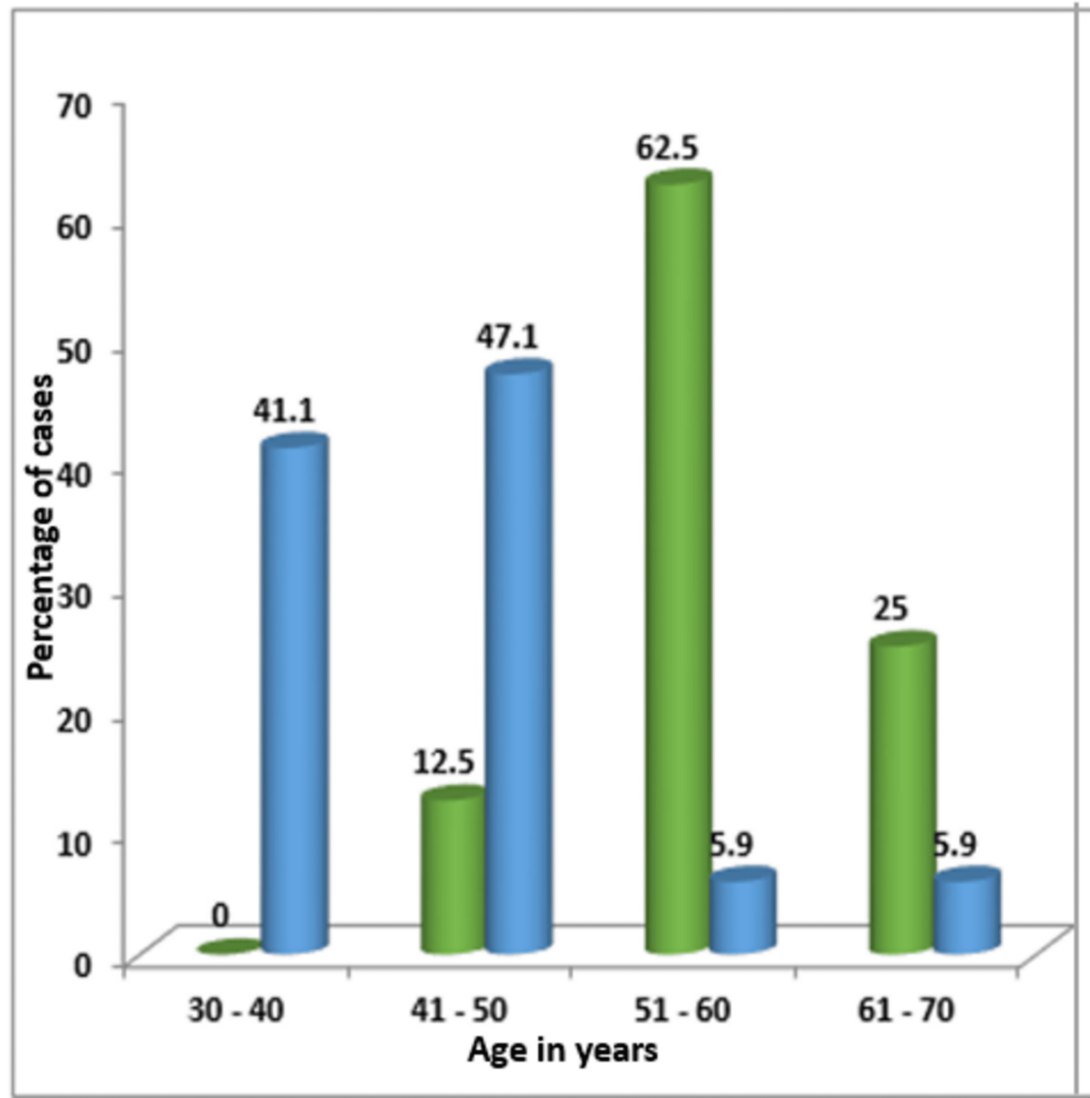
Agewise distribution of endometrial hyperplasia with atypia and without atypia The X axis represents age in years and Y axis represents percentage of cases. The green bar represents atypical hyperplasia and the blue bar represents endometrial hyperplasia without atypia.

The results indicated that all women with EH were aged between 30 and 70 years, with most falling in the 40-50 age range (Figure 1). Women with AH were predominantly in the 51-60 age group, while EH without atypia was most commonly found in women aged 41-50 years. The age distribution for EH without atypia and AH is detailed in Figure 1.

**Figure 2:**
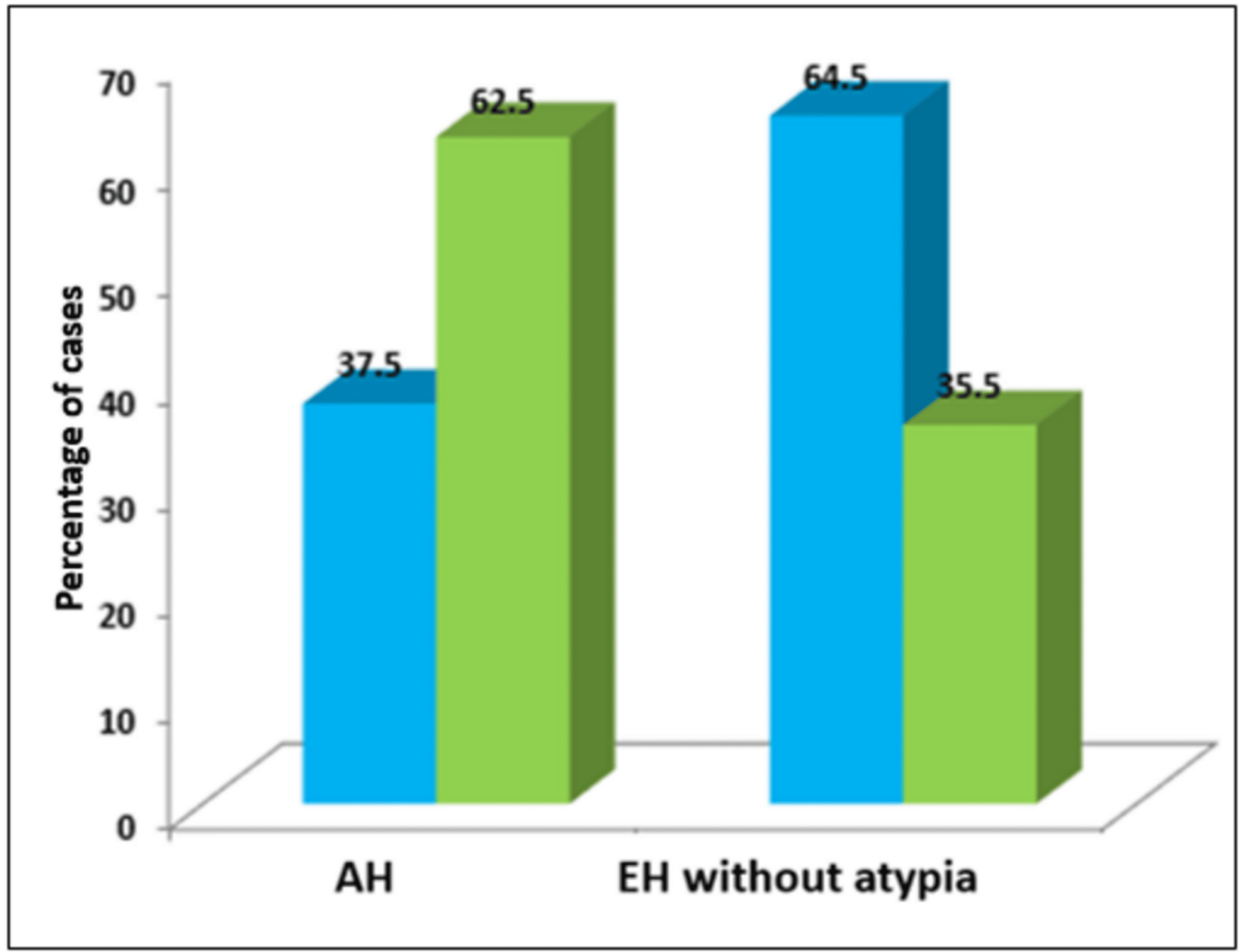
Comparison of presenting symptoms in endometrial hyperplasia (EH) with atypia and without atypia AH, atypical hyperplasia The Y axis represents percentage of cases. X axis - the blue bar represents the number of cases presenting with abnormal uterine bleeding and the green bar represents the number of cases presenting with post-menopausal bleeding.

Most patients with EH exhibited either abnormal uterine bleeding (AUB) or post-menopausal bleeding (PMB) as their primary symptoms. In cases of EH without atypia, AUB was the most common complaint. Since AH was more prevalent in the older age group compared to EH without atypia, PMB was the most frequent presenting symptom. The percentage and distribution of symptoms for EH without atypia and AH are illustrated in Figure 2.

**Figure 3:**
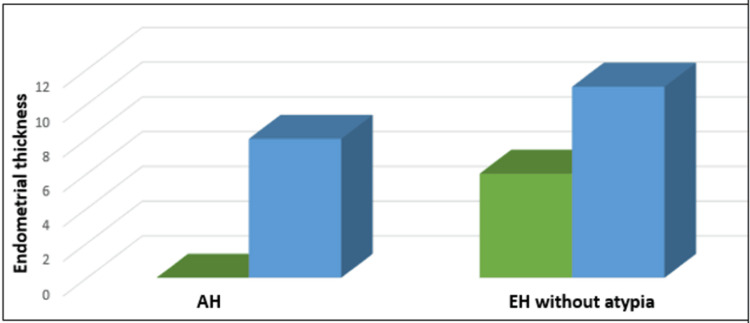
Comparison of endometrial thickness (ET) in patients with endometrial hyperplasia (EH) with atypia and without atypia AH, atypical hyperplasia The Y axis represents endometrial thickness. X axis - the green bar represents ET less than 10 mm and the blue bar represents ET more than 10 mm.

The endometrial thickness in patients with hyperplastic endometrium was compared. All patients with AH had an endometrial thickness exceeding 10 mm. Additionally, over half of the EH without atypia cases also had an endometrial thickness greater than 10 mm. The endometrial thickness for both conditions is depicted in Figure 3.

The p-value for E-cadherin expression in cases of EH without atypia and AH was calculated with the help of a chi-square test. The p-value was 0.05 and was significant (Table 2).

**Table 2:** Comparison of E-cadherin expression in endometrial hyperplasia with atypia and without atypia using the chi-square test *p<0.05, statistically significant

E-cadherin expression	With atypia, N (%)	Without atypia, N (%)	Total, N (%)	χ^2^ value	p-value
Normal	0	05 (29.4)	05 (20)	2.941	0.05*
Downregulated	08 (100)	12 (70.6)	20 (80)		
Total	08 (100)	17 (100)	25 (100)		

**Figure 4:**
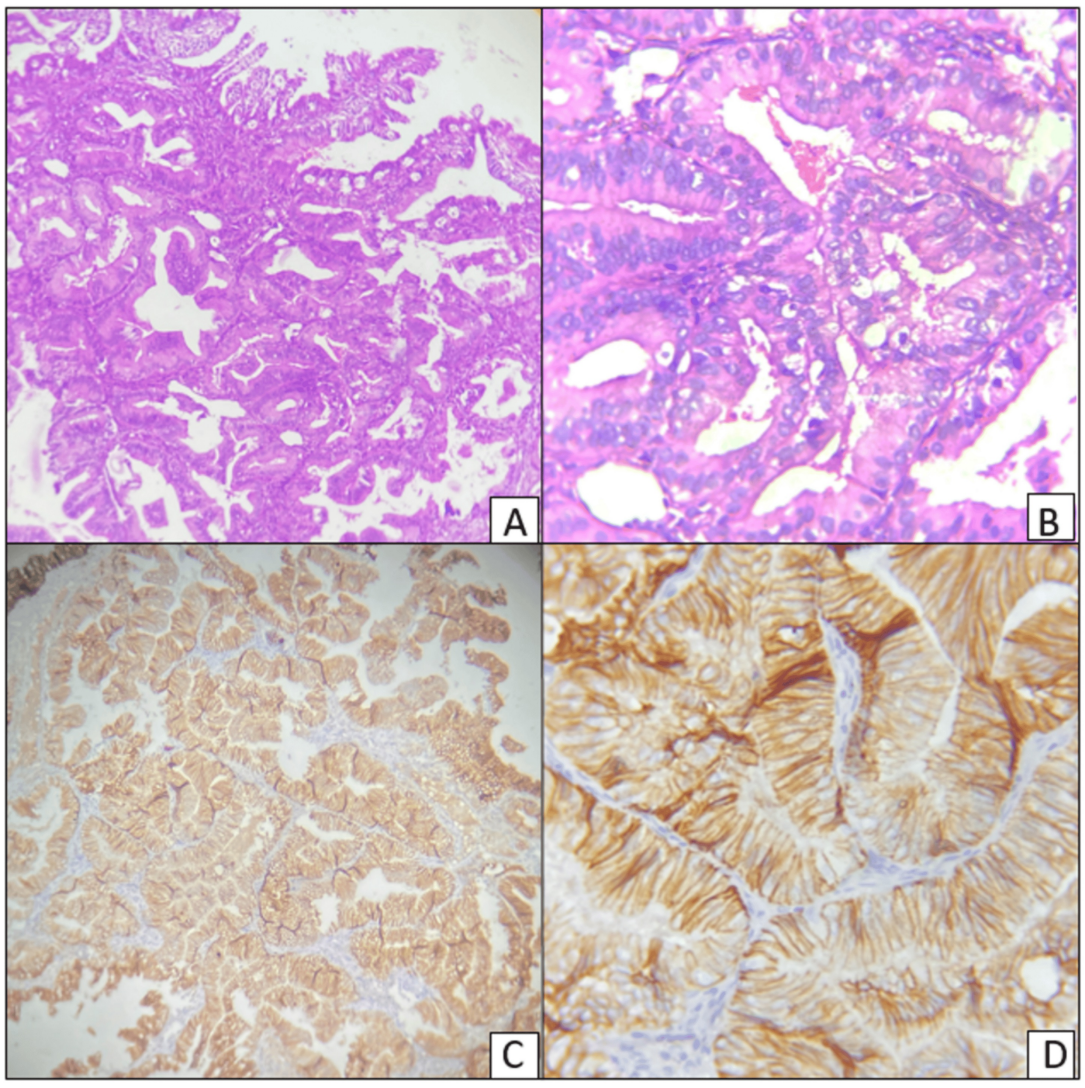
Hematoxylin and eosin-stained sections of atypical hyperplasia of the endometrium (A and B). Immunohistochemical staining of E-cadherin showing weak membrano-cytoplasmic positivity (C and D). Magnification: A and C, 100x; B and D, 400x

In this study of 25 cases of EH, all the eight cases of AH showed heterogeneous or negative staining. No case of AH showed the normal pattern of homogeneous, strong intermembranous staining of E-cadherin. Immunohistochemical staining of AH showing weak membrano-cytoplasmic staining of E-cadherin is shown in Figure 4.

**Figure 5:**
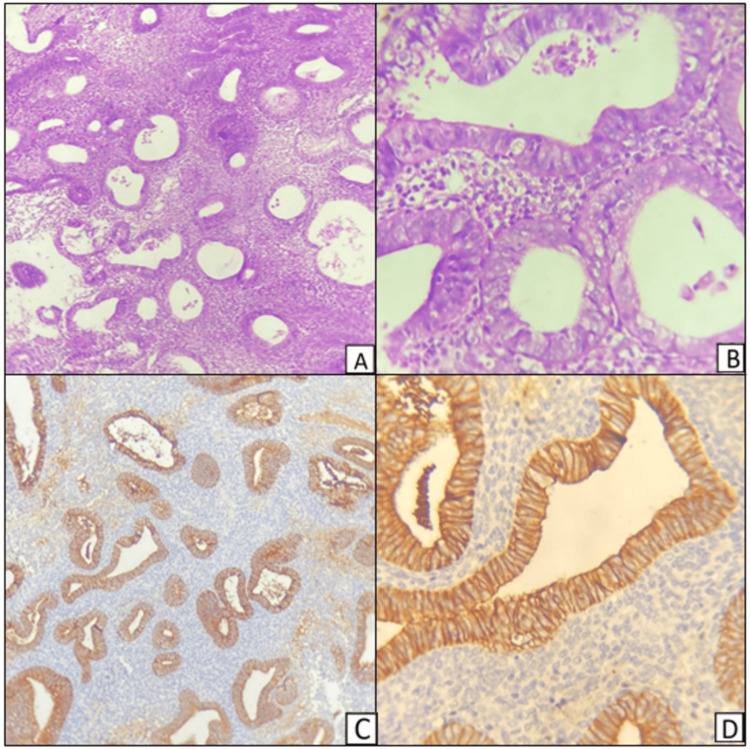
Hematoxylin and eosin-stained sections of endometrial hyperplasia without atypia (A and B). Immunohistochemical staining of the sections showing membrano-cytoplasmic staining of E-cadherin (C and D). Magnification: A and C, 100x; B and D, 400x

**Figure 6:**
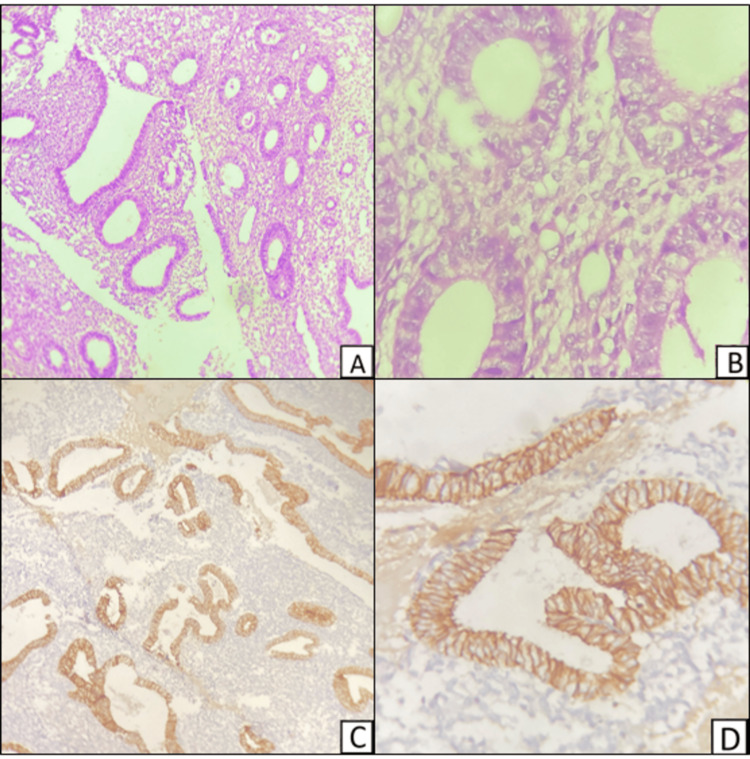
Hematoxylin and eosin-stained sections of endometrial hyperplasia without atypia (A and B). Immunohistochemical staining of the sections show strong membranous positivity for E-cadherin (C and D). Magnification: A and C, 100x; B and D, 400x

Among the cases of EH without atypia, majority of the cases showed heterogeneous staining of E-cadherin (Figure 5) and some cases showed normal membranous staining (Figure 6).

**Figure 7:**
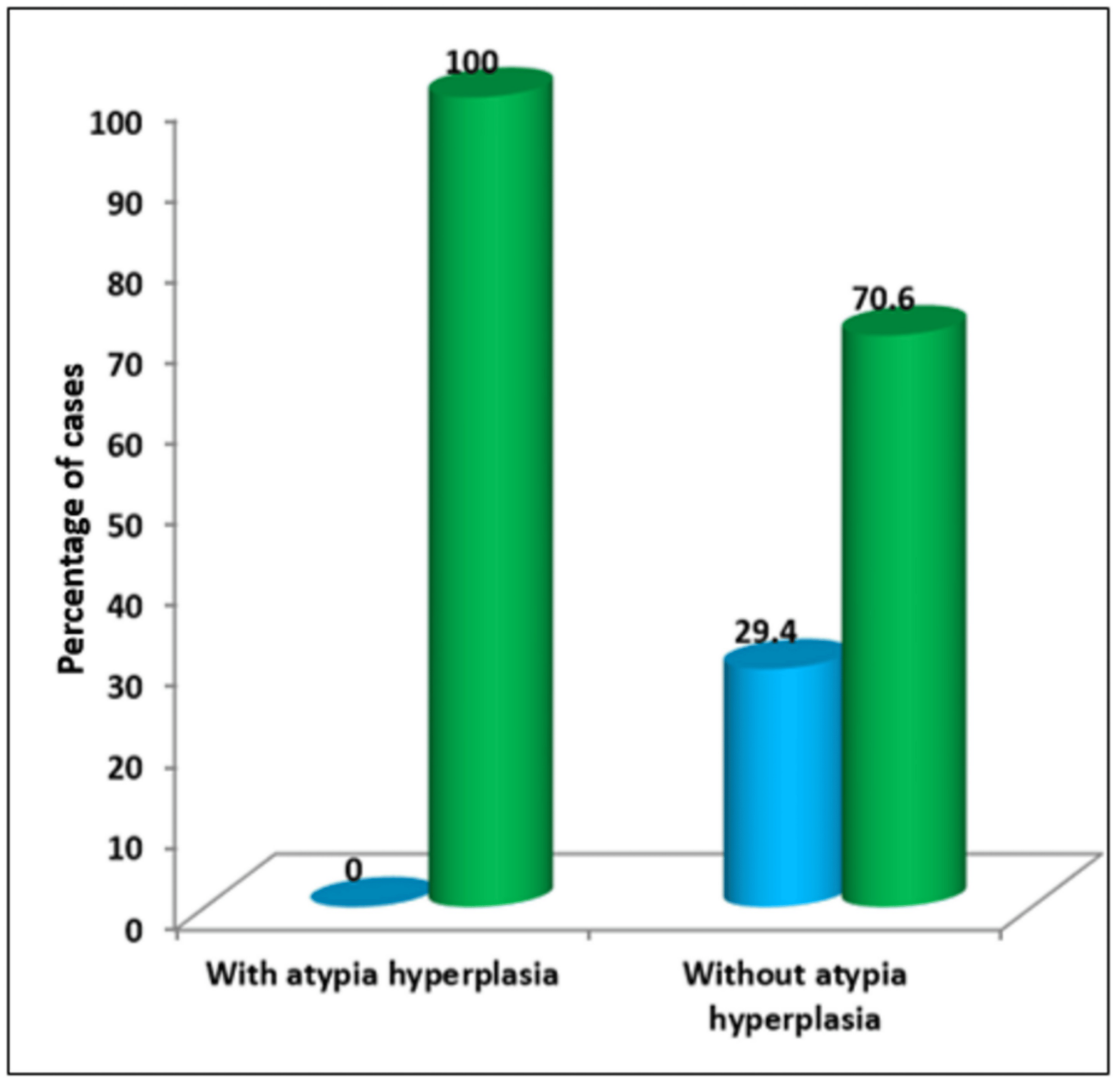
E-cadherin expression in endometrial hyperplasia with atypia and without atypia The Y axis represents the percentage of cases. X axis - the blue bar represents normal expression of E-cadherin and the green bar represents downregulation of E-cadherin

The comparison of E-cadherin expression in both groups is illustrated in Figure 7. 

## Discussion

Adhesion between cells is important to maintain the normal morphological and functional properties of cells in almost all different types of cells, including epithelial, endothelial, and neural tissues. This adhesion is mediated by several cell adhesion molecules called cadherins. By regulating intercellular adhesion, these molecules promote the integrity of the epithelium, thereby contributing to healthy epithelial tissue architecture. There are more than 100 cadherins, classified as classical and non-classical cadherins [1,2]. Some well-known cadherins are E-cadherin and N-cadherin, which interact homotypically, forming adhesion with the same type of cadherins on adjacent cells [3,4]. This adhesion is mediated by calcium-dependent channels. E-cadherin is a transmembrane protein located on the cell membrane. It attaches to the actin cytoskeleton through cytoplasmic domains like α-, β-, and γ-catenins, forming the cadherin-catenin complex. This complex is important for maintaining normal epithelial lining integrity [5,6-8]. Several studies show that malignant tumors exhibit downregulation of E-cadherin with tumor progression, often associated with the invasive and metastatic potential of different tumors [5-8]. There is evidence that E-cadherin plays an important role in the spread and metastasis of many cancers, specifically breast, prostate, gastric, and thyroid carcinomas. E-cadherin down-regulation is often encountered in poorly differentiated tumors and is associated with poor prognosis and survival rate [1,3-7]. E-cadherin/catenin complex dysregulation has been described in several other cancers like carcinoma of the cervix, bladder, esophagus, and colon [7-8]. The presence of E-cadherin is a useful marker for differentiating between ductal carcinoma and lobular carcinoma in the breast. Lobular carcinoma of the breast shows a loss of E-cadherin expression due to loss of the CDH1 gene on chromosome 16. Due to its widespread application in diagnostic pathology and understanding tumor progression, E-cadherin was used in this study to understand its role in endometrial hyperplasia.

The World Health Organization (WHO) and the International Society of Gynecological Pathologists suggested a system for the classification of EH that classified EH in a four-tier system based on gland architecture and cellular atypia [5,6]. Later, in 2014, the WHO simplified the four‑tier system into two groups based on the presence of atypia while ignoring the extent of glandular crowding. EH is currently classified into two broad categories: EH without atypia and endometrial atypical hyperplasia/endometrioid intraepithelial neoplasia (AH/EIN) [1,5]. This distinction is important because clinical management of the two conditions is different, depending on the presence or absence of nuclear atypia [1,6].

EH without atypia is an exaggerated proliferation of glands of irregular size and shape, with an associated increase in the gland-to-stroma ratio compared with proliferative endometrium, but without cytological atypia [1,5]. The associated risk factors for EH include obesity, polycystic ovarian syndrome, and diabetes. EH without atypia results from prolonged estrogen exposure unopposed by progesterone [1,2]. It is mostly diagnosed in perimenopausal women who present with symptoms of abnormal, non-cyclical vaginal bleeding [1]. Grossly, the endometrium is thickened and the thickness varies from 1 to 4 mm. Microscopically, glands vary in size and shape and may be separated by varying amounts of stroma [1,2]. Rarely, they can also show back-to-back crowding of the glands with little intervening stroma; glands are irregularly distributed, creating a variable density of glands in the stroma [1,2]. While some glands may have normal coiled architecture, others can be branched or are cystically dilated. The lining epithelium of the glands is of stratified columnar type, with frequent mitotic figures. Focal hemorrhage and stromal breakdown are common. There is no cytological atypia. Progression to well-differentiated EC occurs in 1%-3% of women with EH without atypia [1,2]. EH without atypia constitutes a benign lesion without significant somatic genetic changes caused by extensive exposure to estrogen that is not counterbalanced by the protective effects of progestins [1,2]. If physiological progesterone levels are resumed or if therapeutic progestins are used, the hyperplastic changes regress and the endometrium becomes healthy again in the majority of cases of EH without atypia [1,2].

Continuous unopposed estrogenic stimulation leads to the progression of EH without atypia to AH/EIN; cytological atypia superimposed on EH defines AH/EIN. The average age at which patients present is 53 years [1,2]. Endogenous or exogenous estrogen exposure is a risk factor. PMB or abnormal vaginal bleeding in perimenopausal women is the most common presenting symptom. AH/EIN coexists with carcinoma in approximately 25%-40% of women. Macroscopically, the gross appearance is variable. The endometrium may be diffusely thickened up to 1 cm and may present as a visible focal thickening resembling a polyp. Microscopically, AH/EIN is composed of crowded aggregates of cytologically altered tubular or branching glands [1,2]. The proportion of glands exceeds that of stroma, resulting in glandular crowding with little intervening stroma [1,2]. The distinction between EH without atypia and AH/EIN is based on nuclear atypia that may include enlargement, pleomorphism, rounding, loss of polarity, and nucleoli. Nuclear atypia is variable [1,2]. There are some molecular alterations identified like microsatellite instability, PTEN mutation and KRAS mutation [1,2]. AH/EIN is often accompanied by metaplastic changes. One-fourth to one-third of women with a biopsy of AH/EIN are diagnosed with cancer at immediate hysterectomy or during the first year of follow-up [1,2].

The percentage of progression to adenocarcinoma is approximately 2% in EH without atypia. According to research, the incidence of progression of EH without atypia to carcinoma is less than 0.4% [8,9]. Conversely, the incidence of carcinoma with AH is about 15%-30%. It was noted that most EH without atypia regressed spontaneously that was not seen in the case of AH [4,8,9]. Several studies suggest a significant overlap between the clinical and histological features of AH and EC in clinical practice as well as in the literature. Therefore, it is reasonable to treat atypical EH as the equivalent of early EC when counselling or treating patients.

The expression of E-cadherin in normal endometrium, that is, in proliferative, secretory phases, and atrophic endometrium has been studied by many researchers. E-cadherin was found to expressed as a homogeneous pattern of immunostaining with intense reactivity at the cell-to-cell borders, whereas a weak immunostaining was present in the cytoplasm [3,8]. No substantial difference in protein expression was evidenced among the different phases of menstrual cycle for normal endometrium [3,8]. No nuclear staining of E-cadherin was observed in glandular cells, and no stromal cells showed any immunoreactivity. Atrophic endometrium cases in postmenopausal women interestingly showed the same pattern for E-cadherin expression [3,8,9]. A homogeneous distribution was seen in all the cases, whereas only 10%-15% cases were negative for E-cadherin. In the case of EH, E-cadherin expression was either homogeneous or heterogeneous and majority of the cases showed heterogeneous expression. Homogeneous expression of E-cadherin was not seen in AH. E-cadherin heterogeneous expression was seen in majority of the cases of AH.

In 2014, Ahmed and Muhammad studied the expression of E-cadherin and CD10 in hyperplastic versus neoplastic endometrium [8]. Their study showed that the mean rank of E-cadherin histoscore in AH was significantly higher than the mean rank in endometrial adenocarcinomas. This shows that the expression of E-cadherin is higher in endometrial adenocarcinomas than in AH of the endometrium [6]. The study also revealed that there is no significant correlation between the stage of the tumor and E-cadherin expression [8]. Carico et al., in 2010, studied the expression of E-cadherin and alpha-catenin in EH and EC [4]. The study included 24 cases of EH without atypia and 11 cases of AH. The study revealed that homogeneous expression of E-cadherin was seen in 58.3% of the cases of EH without atypia. A total of 63% of the cases of AH showed heterogeneous expression of E-cadherin [3]. No case of AH showed homogeneous expression of E-cadherin. Homogeneous E-cadherin expression was decreased in EH without atypia [3]. This shows that loss of E-cadherin is due to cadherin-catenin complex impairment that occurs very early in the hyperplastic process. Yang et al. in 2005 studied the expression of E-cadherin and catenins in EH and carcinoma [10]. This study also showed that there was reduced expression of E-cadherin in AH compared to normal endometrium but the reduction was not as much as with EC [7,8,10].

AH and EC limited within the uterus have a good prognosis and can be surgically cured [10,11]. Tumors that invade the lymphatics or blood vessels and tumors that spread outside the uterus have a poorer prognosis and their treatment usually calls for systemic postoperative adjuvant therapy [12,13]. On a biopsy or curettage specimen, they have similar overlapping features and are almost impossible to differentiate between; there is no differentiating marker to differentiate between the two. In this study, E-cadherin, a well-known epithelial adhesion molecule, showed a heterogeneous membrano-cytoplasmic staining in cases of AH [13-15].

The limitations of this study include a smaller number of cases studied as it was a single-centre study. Although efforts were made to minimize it, there could still be some inter-observer variability in interpreting the immunohistochemical staining results. Another drawback is that these findings should be studied in larger populations with cases of malignancy in the study population to consider E-cadherin as a reliable marker to differentiate between AH and EC.

## Conclusions

E-cadherin expression in cases of EH was evaluated by immunohistochemistry in this study. E-cadherin normally exhibits a strong and pure membranous expression. In cases of an early malignant change in the endometrial glands, due to the impairment of its intercellular adhesion function, E-cadherin shows a membrano-cytoplasmic expression that can help us differentiate between AH and EC. This study shows that the disruption of E-cadherin expression that is seen in EC starts very early that is also seen in its precursor lesions, that is, EH. This emphasizes on the need to closely follow up all cases of EH irrespective of age or clinical presentation. Further studies should be encouraged to study the utility of E-cadherin in predicting the transformation of EH to malignancy.

## References

[1] Singh G, Cue L, Puckett Y (2024). Endometrial hyperplasia. StatPearls [Internet].

[2] Nees LK, Heublein S, Steinmacher S, Juhasz-Böss I, Brucker S, Tempfer CB, Wallwiener M (2022). Endometrial hyperplasia as a risk factor of endometrial cancer. Arch Gynecol Obstet.

[3] Mills AM, Longacre TA (2011). Atypical endometrial hyperplasia and well differentiated endometrioid adenocarcinoma of the uterine corpus. Surg Pathol Clin.

[4] Carico E, Atlante M, Giarnieri E, Raffa S, Bucci B, Giovagnoli MR, Vecchione A (2010). E-cadherin and alpha-catenin expression in normal, hyperplastic and neoplastic endometrium. Anticancer Res.

[5] Yalta T, Atay L, Atalay F, Caydere M, Gonultas M, Ustun H (2009). E-cadherin expression in endometrial malignancies: comparison between endometrioid and non-endometrioid carcinomas. J Int Med Res.

[6] van Roy F, Berx G (2008). The cell-cell adhesion molecule E-cadherin. Cell Mol Life Sci.

[7] Lewczuk Ł, Pryczynicz A, Guzińska-Ustymowicz K (2021). Expression level of E-, N- and P-cadherin proteins in endometrial cancer. Oncol Lett.

[8] Ahmed AR, Muhammad EM (2014). E-cadherin and CD10 expression in atypical hyperplastic and malignant endometrial lesions. J Egypt Natl Canc Inst.

[9] Youssef MY, Mohamed MA (2019). Could E-cadherin and CD10 expression be used to differentiate between atypical endometrial hyperplasia and endometrial carcinoma?. Int J Gynecol Pathol.

[10] Yang YJ, Lee HJ, Lee KH (2005). Expression of E-cadherin and catenin (alpha-, beta-catenin) in endometrial cancer and atypical complex endometrial hyperplasia. Korean J Obstet Gynecol.

[11] Robbe EJ, van Kuijk SM, de Boed EM, Smits LJ, van der Wurff AA, Kruitwagen RF, Pijnenborg JM (2012). Predicting the coexistence of an endometrial adenocarcinoma in the presence of atypical complex hyperplasia: immunohistochemical analysis of endometrial samples. Int J Gynecol Cancer.

[12] Shaco-Levy R, Sharabi S, Piura B, Sion-Vardy N (2008). MMP-2, TIMP-1, E-cadherin, and beta-catenin expression in endometrial serous carcinoma compared with low-grade endometrial endometrioid carcinoma and proliferative endometrium. Acta Obstet Gynecol Scand.

[13] Scholten AN, Aliredjo R, Creutzberg CL, Smit VT (2006). Combined E-cadherin, alpha-catenin, and beta-catenin expression is a favorable prognostic factor in endometrial carcinoma. Int J Gynecol Cancer.

[14] Sakuragi N, Nishiya M, Ikeda K (1994). Decreased E-cadherin expression in endometrial carcinoma is associated with tumor dedifferentiation and deep myometrial invasion. Gynecol Oncol.

[15] Moreno-Bueno G, Hardisson D, Sarrió D (2003). Abnormalities of E- and P-cadherin and catenin (beta-, gamma-catenin, and p120ctn) expression in endometrial cancer and endometrial atypical hyperplasia. J Pathol.

